# Molecular Characterization of Voltage-Gated Sodium Channels and Their Relations with Paralytic Shellfish Toxin Bioaccumulation in the Pacific Oyster C*rassostrea gigas*

**DOI:** 10.3390/md15010021

**Published:** 2017-01-19

**Authors:** Floriane Boullot, Justine Castrec, Adeline Bidault, Natanael Dantas, Laura Payton, Mickael Perrigault, Damien Tran, Zouher Amzil, Pierre Boudry, Philippe Soudant, Hélène Hégaret, Caroline Fabioux

**Affiliations:** 1 Laboratoire des Sciences de l’Environnement Marin (LEMAR), Institut Universitaire Européen de la Mer, Université de Bretagne Occidentale, UMR 6539 CNRS/UBO/IRD/Ifremer, 29280 Plouzané, France; justine.castrec@univ-brest.fr (J.C.); adeline.bidault@univ-brest.fr (A.B.); philippe.soudant@univ-brest.fr (P.S.); helene.hegaret@univ-brest.fr (H.H.); 2 Laboratory of Immunology and Pathology of Invertebrates, Department of Molecular Biology, Exact and Natural Sciences Center, Federal University of Paraíba—Campus I, 58051-900 João Pessoa, PB, Brazil; natan.cbio@gmail.com; 3 UMR 5805 EPOC, CNRS—Équipe Écotoxicologie Aquatique, Université de Bordeaux, Station Marine d’Arcachon, 33120 Arcachon, France; l.payton@epoc.u-bordeaux1.fr (L.P.); mickael.perrigault@u-bordeaux.fr (M.P.); d.tran@epoc.u-bordeaux1.fr (D.T.); 4 Laboratoire Phycotoxines, IFREMER, BP 21105, 44311 Nantes, France; zouher.amzil@ifremer.fr; 5 Ifremer, UMR 6539 LEMAR CNRS/UBO/IRD/Ifremer, 29280 Plouzané, France; pierre.boudry@ifremer.fr

**Keywords:** *Crassostrea gigas*, sodium channel, alternative splicing, *Alexandrium minutum*, paralytic shellfish toxins

## Abstract

Paralytic shellfish toxins (PST) bind to voltage-gated sodium channels (Nav) and block conduction of action potential in excitable cells. This study aimed to (i) characterize Nav sequences in *Crassostrea gigas* and (ii) investigate a putative relation between Nav and PST-bioaccumulation in oysters. The phylogenetic analysis highlighted two types of Nav in *C. gigas*: a Nav1 (*CgNav1*) and a Nav2 (*CgNav2*) with sequence properties of sodium-selective and sodium/calcium-selective channels, respectively. Three alternative splice transcripts of *CgNav1* named A, B and C, were characterized. The expression of *CgNav1*, analyzed by in situ hybridization, is specific to nervous cells and to structures corresponding to neuromuscular junctions. Real-time PCR analyses showed a strong expression of *CgNav1A* in the striated muscle while *CgNav1B* is mainly expressed in visceral ganglia. *CgNav1C* expression is ubiquitous. The PST binding site (domain II) of *CgNav1* variants possess an amino acid Q that could potentially confer a partial saxitoxin (STX)-resistance to the channel. The *CgNav1* genotype or alternative splicing would not be the key point determining PST bioaccumulation level in oysters.

## 1. Introduction

Phycotoxins are natural compounds produced by phytoplanktonic species that can be responsible for many human illnesses and poisoning linked to contaminated seafood consumption. With favorable environmental conditions, toxic microalgae can proliferate and aggregate to form harmful algal blooms (HAB). These natural phenomena have increased in recent years, both in frequency and in a worldwide geographical distribution [1]. The HAB are a major health risk [2], can cause economic losses associated with fishery or aquaculture closure and sale prohibition, and can have ecological consequences on marine ecosystems [3,4]. Paralytic shellfish poisoning (PSP) is one of the highest threats to human health among poisoning by phycotoxins. The toxins involved in this syndrome are the paralytic shellfish toxins (PST), produced by dinoflagellates, mainly of the genus *Alexandrium*.

Suspension-feeders such as bivalve molluscs that consume phytoplankton can bioaccumulate large amounts of toxins during these blooms. Consumption of PST-contaminated shellfish represents one of the main vectors of PSP illnesses in humans. In Europe, shellfish toxin content is subjected to health regulations defined by the European Union (CE Regulation No. 854/2004), which prohibits the harvesting and sale of shellfish with more than 80 μg of STX equivalent to 100 g of shellfish meat. PST are composed of many toxic derivatives of saxitoxin (STX), the most potent toxin. STX and its derivatives bind to the voltage-gated sodium channels (Nav) and block conduction of action potential. The Nav channel plays a crucial role in membrane excitability in nerve cells, which makes it the target of many neurotoxins produced by animals or plants, such as STX and tetrodotoxin (TTX).

**Figure 1:**
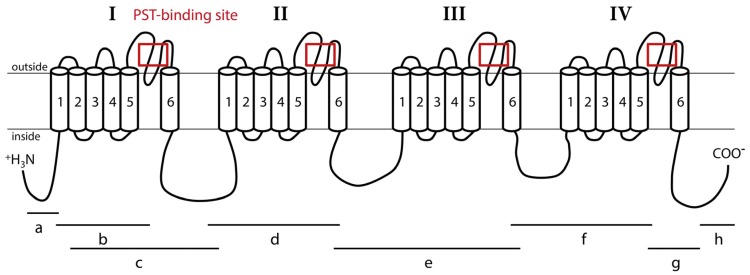
Representation of the Nav1 α subunit of *C. gigas* oysters. This channel is composed of four homologous domains (I–IV), each having six transmembrane segments (1–6). Fragments “a”, “b”, “c”, “d”, “e”, “f”, “g” and “h” were used to obtain the complete sequence of the Nav. Lines indicate the location of PCR amplicons relative to the channel structure. Red boxes indicate sequenced regions including paralytic shellfish toxin (PST) binding site used for the study of Nav genomic polymorphism.

Nav channels are large and complex transmembrane proteins responsible for electrical excitability of cells [5]. Increased sodium permeability induces membrane depolarization, producing action potentials and electrical conduction [6]. Nav channels are composed of a main α subunit, responsible for the selectivity of the channel and an accessory subunit that can complete functions of the Nav channel. Expression of the α subunit alone may be sufficient to produce sodium currents in the heterologous system. Two types of genes encoding the α subunit Nav channel exist in invertebrates (coding for Nav1 and Nav2); whereas, vertebrates possess at least nine α subunit Nav genes, all coding for the Nav1 channel family [7]. Accessory subunits are called β subunit in mammals, and have homologous in other vertebrates [8,9]. Accessory subunits have been characterized in insects with a tipE subunit in *Drosophila*, and tipE-homologous in other insects [10,11]. The β subunits modulate gating and membrane expression of Nav channels [12]. The α subunit consists of 4 homologous domains (I–IV) (Figure 1). Each domain has 6 transmembrane segments (S1–S6) connected by intra- and extra-cellular loops [13,14,15]. The S4 segments are charged positively and are responsible for voltage sensitivity [6,14]. The loop between S5 and S6 segments forms the ion-selectivity filter and is named pore-loop or P segment [16]. The selectivity filter contains a specific pattern of amino acids selective for sodium ions only: D400 (of rat Nav1.4) in domain I, E755 in domain II, K1237 in domain III, and A1529 in domain IV [17]. STX, like TTX, is known to bind at the P segment, or site 1, of Nav [6,14].

In the softshell clam, *Mya arenaria*, on the eastern coast of North America, two populations were studied by Bricelj et al. [18]: one of them, regularly exposed to bloom of *Alexandrium* spp., was proved to be resistant to PST effects upon neuromuscular impairment, clams accumulating high levels of PST without dying. The other population, never exposed to *Alexandrium* blooms, was referred to as sensitive because of the death of experimentally-exposed individuals [18]. A polymorphism of a single amino acid at the STX binding site of the α subunit Nav sequence was shown to be associated with decreased affinity for STX up to 1000-fold, explaining the lower nerve sensitivity and the higher PST accumulation in resistant populations of softshell clams exposed to *Alexandrium* spp. compared to the sensitive population [4]. Alternatively, post-transcriptional regulations also can generate molecular diversity in Nav α subunit and have been associated with contrasting phenotypes of Nav sensitivity to neurotoxins. In insects, alternative splicing leads to the formation of two distinct variants of para channels (the Nav1 channels of insects) with different sensitivity to a pyrethroid insecticide, one being 100-fold less sensitive to insecticide than the second, as demonstrated in the German cockroach *Blattella germanica* [19]. In the Pacific oyster, *Crassostrea gigas*, high inter-individual variability in PST bioaccumulation was measured in oysters exposed to a toxic strain of *A. minutum* [20]. This variability could originate from physiological plasticity of oysters, for example from a variation of feeding behaviour between oysters as proposed by Haberkorn et al. [20], and/or different sensitivities of the voltage-gated sodium channel.

The present study investigated the implication of the voltage-gated sodium channel and its potential isoforms in the variability of PST accumulation in oysters. First, the analysis of the two sequences of Nav α subunit available in the *C. gigas* genome database (annotated Nav5 and Nav9) [21], allowed determination of the phylogenetic status of Nav genes in different invertebrate species. Nav9 and Nav5 were proposed to be renamed *CgNav1* and *CgNav2*, respectively, in accordance with phylogenetic positioning. Then, the study focused only on *CgNav1*, assessing the expression of *CgNav1* α subunit by in situ hybridization and real-time PCR in all tissues of oysters. The existence of *CgNav1* isoforms, either resulting from genetic polymorphism or from alternative mRNA splicing, was also investigated. Three alternative splice variants were characterized. Finally, we examined the link between PST accumulation and the expression of splice variants in oysters experimentally exposed to *A. minutum*.

## 2. Results

### 2.1. Phylogenetic Analysis of Nav Channels in *Crassostrea gigas*

**Figure 2:**
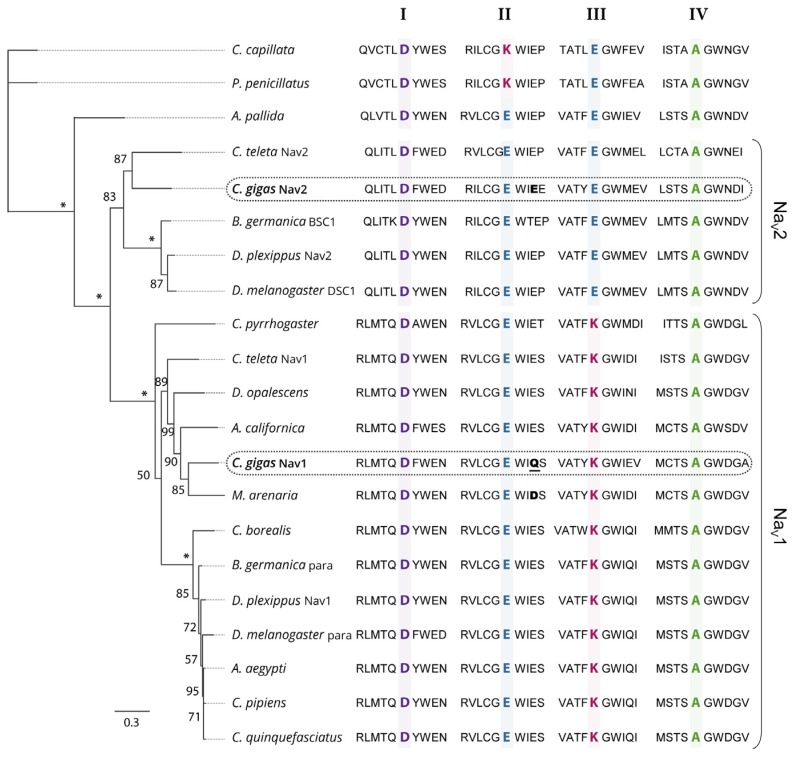
Maximum likelihood phylogenetic tree of the voltage-gated sodium channel α subunit family. The ten amino acids of the selectivity filter, in each domain (I–IV), are represented and key amino acids for the selectivity in each domain (DKEA, DEEA, and DEKA) are highlighted and bold. The amino acid responsible of the sensitivity to STX is bold for *CgNav2* and *M. arenaria* Nav and bold and underlined for *CgNav1*. The numbers indicate the bootstraps score for 100 replications, with stars indicating scores of 100%. Species used for the phylogenetic analysis and the associated accession number of the Nav sequence: *Cyanea capillata* (AAA75572), *Polyorchis penicillatus* (AAC09306), *Aiptasia pallida* (AAB96953), *Capitella teleta* Nav2 (JGI protein ID 134859), *Crassostrea gigas* Nav2 (EKC21550), *Blattella germanica* BSC1 (AAK01090), *Danaus plexippus* Nav2 (EHJ64356), *Drosophila melanogaster* DSC1 (ABF70206), *Capitella teleta* Nav1 (JGI protein ID 210954), *Cynops pyrrhogaster* (AAD17315), *Doryteuthis opalescens* (AAA16202A), *Aplysia californica* (NP_001191637), *Mya arenaria* (AAX14719), *Crassostrea gigas* Nav1 (EKC22630), *Cancer borealis* (ABL10360), *Blattella germanica* para (AAC47483), *Danaus plexippus* Nav1 (EHJ74501), *Drosophila melanogaster* para (AAB59195), *Aedes aegypti* (ACB37023), *Culex pipiens* (AGO33659), *Culex quinquefasciatus* (AGO33660) (See Table 4 for references).

Phylogenetic relationships between the protein sequences of Nav α subunit characterized in *C. gigas* (EKC22630 and EKC21550) and the Nav previously reported in other invertebrate species were studied (Figure 2). Analyses revealed that sequences annotated Nav9 (EKC22630) and Nav5 (EKC21550) channels in *C. gigas* did not cluster together but branched at the root defining the two clades of Nav channels (Nav1 and Nav2): the Nav9 sequence appeared grouped with Nav1 channels, but Nav5 grouped with Nav2. Accordingly, we renamed Nav9 as *CgNav1* and Nav5 as *CgNav2*. The Nav1 cluster includes para type channel and Nav2 includes BSC1/DSC1 type channel found in insects. The Pacific oyster Nav1 channel showed a very close phylogenetic relationship with the Nav channel of the clam *M. arenaria* and with para-like channels characterized in other mollusc species: in the gastropod *Aplysia californica* and in the cephalopod *Doryteuthis opalescens*. Contrastingly, *C. gigas* Nav2 channel presented a close relationship with the Nav2 channel of *Capitella teleta*, BSC1 and DSC1 channels of the insects *Blatella germanica* and *Drosophila melanogaster*, respectively. To our knowledge, *CgNav2* is the first member of Nav2 cluster characterized in bivalves. Analyses of the selectivity filters revealed a DEKA motif for the Nav1 of *C. gigas* as observed in all Nav1 channels. This sequence gives the channel selectivity to sodium ions only. For the Nav2 of *C. gigas*, a DEEA motif was revealed as observed in the Nav2 channels of anthozoans and bilaterians. These results suggest that the Nav2 channel is likely to be selective to sodium and calcium ions. Considering these results, only the *CgNav1* channel was considered for further characterization steps in the study of relationships between paralytic shellfish toxins accumulation and sodium channel characteristics, as the sodium channel is known to be the first target of PST.

### 2.2. Structure of CgNav1 α Subunit

**Figure 3:**
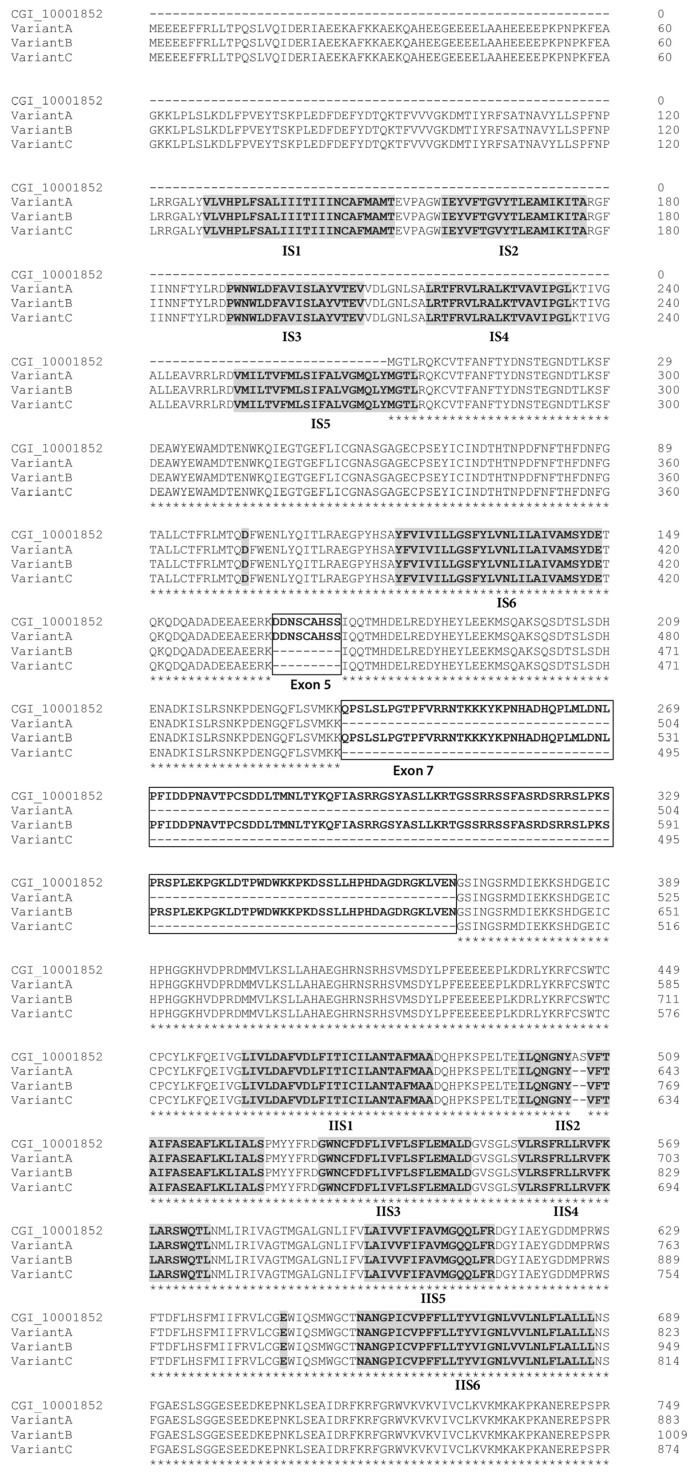

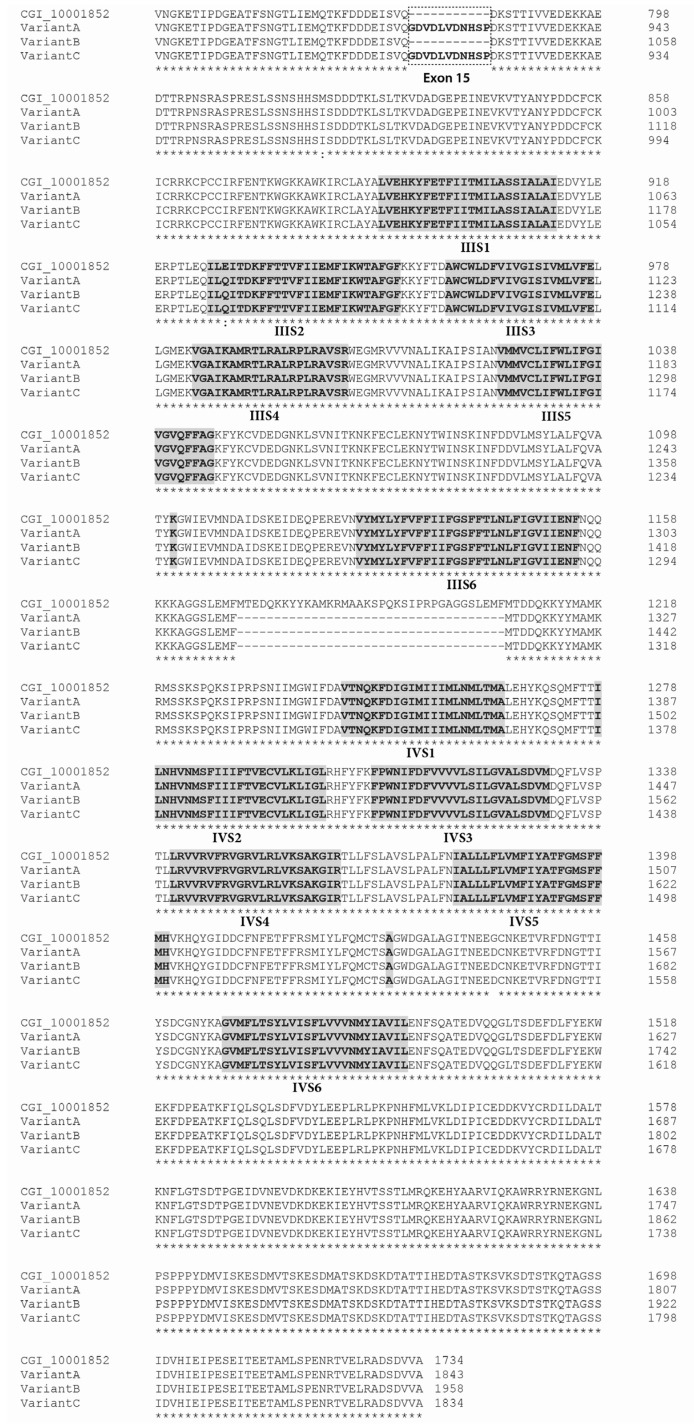
Alignment of *CgNav1* α subunit protein sequences: the reference sequence CGI_10001852, variant A, variant B and variant C. The four homologous domains are annotated I, II, III and IV. The transmembrane segments (S1–S6) are highlighted in black. The motif DEKA responsible of selectivity for sodium ions, in S5–S6 linker, is highlighted in black. The spliced exons are boxed with bold line. The alternate exon 15 is boxed with dotted lines. Stars indicate shared amino acids.

The *CgNav1* α subunit was amplified and sequenced step by step using 8 overlapping cDNA fragments covering the entire open reading frame (ORF) (Figure 1). The 8 overlapping fragments correspond to: 575 bp from 5′UTR to segment 1 of domain I (IS1), 444 bp from IS1 to IS5-IS6, 1813 bp from IS1 to IS6-IIS1, 904 bp from IS6-IIS1 to IIS6-IIIS1, 1337 bp from IIS6-IIIS1 to IIIS6-IVS1, 736 bp from IIIS6 to IVS5-IVS6, 724 bp from IVS5-IVS6 to stop codon and 533 bp from stop codon to 3′UTR. The consensus sequence created using Geneious software has been compared to the Na_V_9 α subunit genomic reference sequence (CGI_10001852). This allowed the identification of 25 exons (including an alternate exon, see Section 2.4) and 24 introns (Figure 3). The exons were numbered from 1 to 25. The size of exons ranges from 27 bp to 1235 bp and the size of introns ranges from 89 bp to 2389 bp.

### 2.3. *CgNav1* DNA Polymorphism

The polymorphism was investigated in the DNA sequences of the region surrounding the PST binding sites of the 4 domains (I, II, III, and IV) of *CgNav1* α subunit. The size of amplicons was 428 bp, 136 bp, 328 bp and 301 bp for domains I, II, III and IV, respectively. In the 644 sequences analysed (4 regions sequenced per gene, analysed on 161 oysters sampled from all 4 populations) only 3 non-synonymous polymorphisms were identified. These are located outside of the 10 amino acids constituting the PST binding sites. Thus, the protein sequence of the PST binding site appeared perfectly conserved in all the individuals analysed. The number of SNP (Single Nucleotid Polymorphism), calculated per population and per domain of the *CgNav1* α subunit, varied from 0 to 5 in exonic regions and 2 to 7 in intronic zones (Table 1). The level of SNP was 1/61 bp in coding regions and 1/40 bp in non-coding regions. Global genetic diversity calculated as the mean ∏t of the 4 domains was similar in all populations (∏t mean = 0.016).

### 2.4. Identification of *CgNav1* Splice Variants

The cDNA fragments “a” to “h” (Figure 1) were individually amplified in 9 tissues of 5 oysters, to reveal potential splice variants. Splice variants were discriminated on the basis of size using electrophoresis. The fragment “c” (from 361 base pair (bp) to 2234 bp length) was the unique amplicon presenting size variations between samples. Sequencing analyses and alignment of *CgNav1* cDNA to CGI_10001852 revealed the existence of three different splice variants named A, B and C. The alignment of variant sequences on the CGI_10001852 reference sequence allowed determination of which exons were spliced (Figure 3). Sequences were deposited in GenBank with accession numbers KY020155, KY020156 and KY020157 for variant A, B and C, respectively.

**Figure 4:**
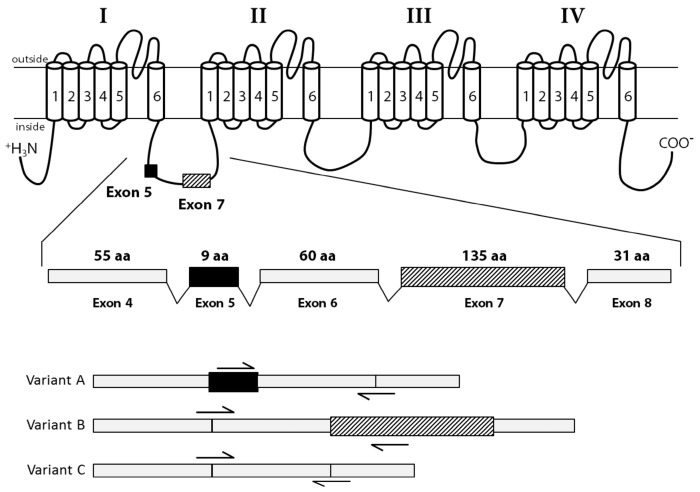
Alternative splicing of the *C. gigas* Nav1 α subunit. This channel is composed of four homologous domains (I–IV), each having six transmembrane segments (1–6). Location of alternatively spliced exons is noted on the protein structure with boxes. Detail of the mRNA structure is provided, where two alternative exons were found (exons 5 and 7), resulting in 3 splice isoforms (variants A, B and C). Variants-specific primers were represented by arrows.

Variant A lacks exon 7 (135 amino acids), which encodes a region of the intracellular inter-domain between domain I and II (ID I-II) rich in proline and serine residues. Variant B lacks exon 5 (9 amino acids), which also encodes a part of the ID I-II. Finally, variant C lacks both 5 and 7 exons. Exons 5 and 7 seem to be mutually exclusive exons, as no complete sequence with both exons has been found. Variants A and C also have retained a part of the intron 14 (33 bp), that could correspond to an alternate exon, which is predicted to encode a part of the intracellular ID II-III. We named this alternate exon “15”. All the characteristics of transcript variants are summarized in Table 2 and Figure 4.

Analyses of the predicted protein structure of splice variants showed that exon 5 has three serine residues, which are polar amino acids and could be phosphorylated. Exon 7 has many important residues. There are 15 proline residues at the beginning and the end of the exon and 17 serine residues in the middle of the exon, all corresponding to many phosphorylation sites. The alternate exon 15, which is present in variants A and C, had only one proline residue. These results were confirmed by the prediction of the phosphorylated sites in both exons. Exon 5 has two protein kinase C (PKC) predicted sites; whereas, exon 7 had 12 protein kinase A (PKA) predicted sites.

### 2.5. Tissue-Level CgNav1 α Subunit Expression Patterns

**Figure 5:**
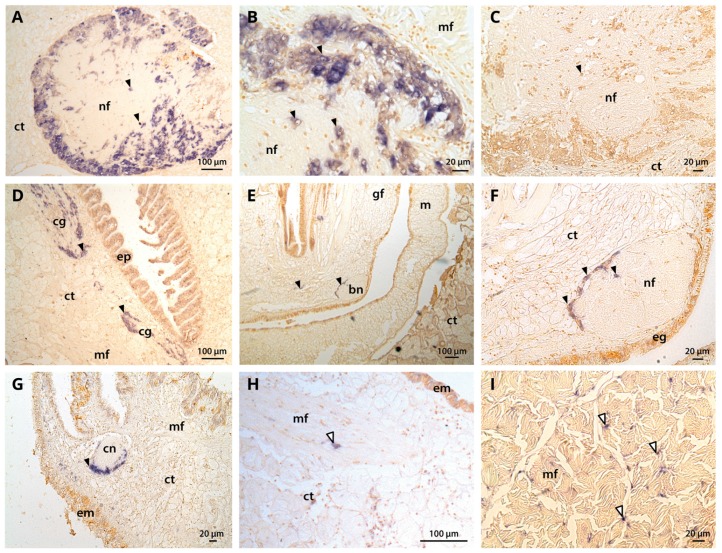
Tissue localization of the *C. gigas* Nav1 α subunit by in situ hybridization using DIG labelling. (**A**,**B**) nerve cells and nerve fibres constituting the visceral ganglion; (**C**) negative control for visceral ganglion; (**D**) cerebral ganglion located at the base of the labial palps; (**E**,**F**) branchial nerve at the base of the gills; (**G**) circumpallial nerve in the mantle edge; (**H**) neuromuscular junction in the mantle; (**I**) neuromuscular junctions and muscle fibres in striated muscle. Black arrow head corresponds to nerve cells (A, B, C, D, E, F, and G) and white arrow head to neuromuscular junctions (H and I) containing *CgNav1* mRNA. ct: connective tissue, nf: nerve fibres, mf: muscle fibres, cg: cerebral ganglion, ep: epithelial cells of labial palps, bn: branchial nerve, gf: gills filaments, m: mantle, eg: epithelial cells of gills, em: epithelial cells of mantle, cn: circumpallial nerve.

Expression of *CgNav1* channel was investigated by real-time PCR and by in situ hybridization (Figure 5) using primers amplifying a sequence common to all the variants. Real-time PCR analyses showed that *CgNav1* gene appeared predominantly expressed in visceral ganglia (relative quantification, Qr = 14). *CgNav1* is also expressed in striated muscle (Qr = 3.2). The expression of *CgNav1* in the gills (Qr = 2.3) is twice as intense as in the mantle (Qr = 1) and four times higher than in the labial palps (Qr = 0.5). The *CgNav1* gene is expressed less in the smooth muscle (Qr = 0.3) and almost absent in gonad (Qr = 0.2), heart, and digestive gland (Qr = 0.1 for both).

In situ hybridization showed that *CgNav1* is selectively expressed in the nerve cells of the visceral ganglia, located on the peryphery of the ganglia (Figure 5A–C). *CgNav1* mRNA were also detected in the nerve cells of cerebral ganglia at the base of the labial palps but not in the adjacent connective tissue (Figure 5D). As in visceral ganglia, nerve cells are located on the periphery of the cerebral ganglia but seem less abundant. Clear staining was detected in the nerve cells of the branchial nerve near the gill axis (Figure 5E,F), and in the nerve cells of the circumpallial nerve which runs along the mantle edge Figure 5G). Staining spots of 5–6 μm-size were observed abundantly among the muscle fibres of the striated muscle and sporadically in mantle (Figure 5H); in both tissues, the staining was not localized in a delimitated cell structure (Figure 5I). According to the description of the adductor muscle of the eastern oyster by Morrison [22], this staining could be localized in the nerve ending that often occurr close to the sarcolemma, corresponding to neuromuscular junctions. No signal was observed in the smooth muscle, neither inside the muscle fibres themselves nor in the nerve fibres. In all tissues, the observed staining of *CgNav1* mRNA corresponds to the nerve cell bodies or the neuromuscular junctions but not to the axons of the neurons.

### 2.6. Expression Patterns of *CgNav1* α Subunit Splice Variants

**Figure 6:**
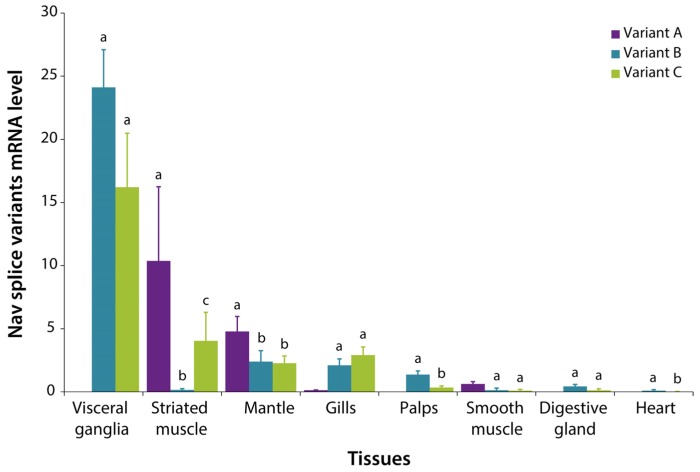
Expression of *CgNav1* α subunit splice variants A, B and C related to *EF1α* and *GAPDH* genes measured by real-time PCR in tissues of *C. gigas* oyster. Relative quantity of Nav transcripts was calculated according to the E-method and using the mean of the two reference genes (Roche). Letters show significant differences between expression patterns of splice variants within the tissue. Homogeneous groups share letters.

The expression of the splice variants A, B and C of *CgNav1* α subunit was examined by real-time PCR using variant-specific primers (Table 3 and Figure 6). Variant A is most expressed in striated muscle (Qr = 10.4), then in decreasing order, in mantle (Qr = 4.8), smooth muscle (Qr = 0.6), gills (Qr = 0.1), and no expression was detected in visceral ganglia, labial palps, digestive gland, or heart. Variant B is mainly expressed in visceral ganglia (Qr = 24.1) and weakly expressed, in decreasing order, in mantle (Qr = 2.4) (inter-organ comparisons, *n* = 5, *p <* 0.001), gills (Qr = 2.1), labial palps (Qr = 1.4) (*n* = 5, *p* = 0.016), digestive gland (Qr = 0.4), striated muscle (Qr = 0.2) (*n* = 5, *p* = 0.01), smooth muscle (Qr = 0.1) and heart (Qr = 0.1). Variant C also is highly expressed in visceral ganglia (Qr = 16.2), then more weakly in striated muscle (Qr = 4) (*n* = 4, *p* = 0.009), gills (Qr = 2.9), mantle (Qr = 2.3), labial palps (Qr = 0.4) (*n* = 6, *p <* 0.001), digestive gland (Qr = 0.1), smooth muscle (Qr = 0.1), and almost not expressed in heart (Qr = 0.02). These results indicate that variant A (with exon 5) is never expressed in visceral ganglia, digestive gland, or labial palps. Variant B (with exon 7) is almost never expressed in striated muscle.

### 2.7. Relationship between Expression of *CgNav1* α Subunit Splice Variants and PST Accumulation

**Figure 7:**
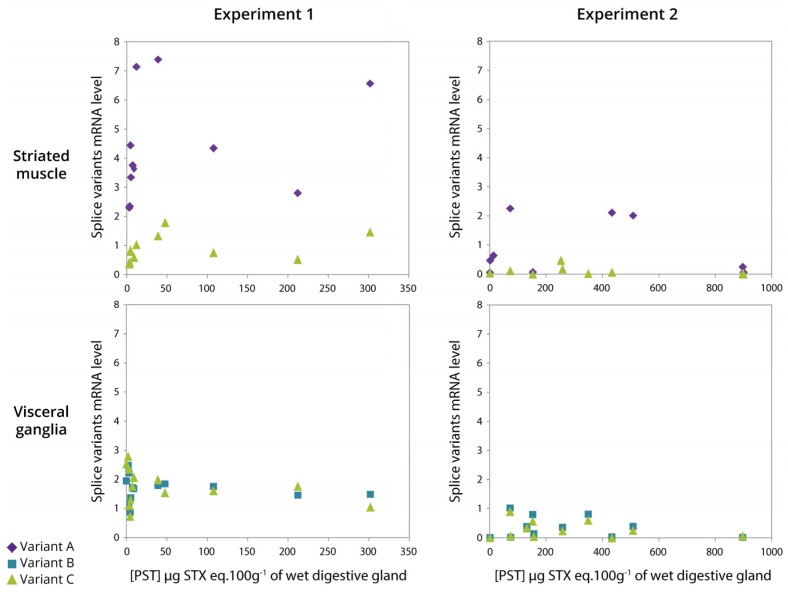
Relationship between *CgNav1* splice variants mRNA levels in striated muscle and visceral ganglia and PST accumulation in digestive gland of *C. gigas* oyster experimentally exposed to *Alexandrium minutum* (**Experiments** **1** and **2**).

PST accumulation in the digestive glands of oysters exposed to *A. minutum*, ranged from 2 to 302 (experiment 1) and from 2 to 900 (experiment 2) μg STX eq. 100 g^−1^ of wet digestive gland, the organ that accumulated the most (Figure 7). As a result, the toxin content varied by a factor 150 (experiment 1) and 450 (experiment 2) between oysters. According to the results presented in Figure 6, only variants A and C are represented for striated muscle, and variants B and C in visceral ganglia, in both experiments (Figure 7). In the striated muscle, no statistical significant correlation was observed between the expression of *CgNav1* and toxin accumulation when the analyses were made on the full range of toxin accumulation values. However, *CgNav1A* and *CgNav1C* expression tended to increase according to PST accumulation for oysters with low toxin content (<100 μg STX eq. 100 g^−1^ of digestive gland). In visceral ganglia, a high inter-individual variability of the expression levels of variants B and C were observed for oysters that have very low toxin content. No correlation was observed between toxin content and expression in this tissue.

## 3. Discussion

### 3.1. Two Genes Encoding Two Types of Nav Channels (Nav1 and Nav2) in *C. gigas*

The mains objectives of our study were to characterize the voltage-gated sodium channel α subunit in the Pacific oyster *C. gigas* and explore a potential relationship between expression and PST bioaccumulation. In NCBI databases, two sequences are annotated as Nav genes (*Nav9* and *Nav5*) for *C. gigas* [21]. Our results revealed that *Nav9* and *Nav5* cluster, respectively, with *Nav1*-type and *Nav2*-type genes and we, therefore, proposed to rename the *C. gigas* Nav genes *CgNav1* (*Nav9*, EKC22630) and *CgNav2* (*Nav5*, EKC21550). The sequence EKC22630 appeared incomplete, although it results from the oyster genome sequencing. This underlines the necessity to check different databases or to control candidate sequences by amplification and sequencing to obtain the most accurate reference sequence as highlighted by Rivière et al. [24].

Voltage-gated sodium channels share the amino acid sequence DEKA (for domains I, II, III and IV, respectively) responsible for the selectivity filter of the pore (between S5 and S6 in each domain) [14,25]. Our results showed that the DEKA sequence is found in the *CgNav1* sequence as in the other Nav1-type proteins, but the protein *CgNav2* presents a DEEA sequence characteristic of Nav2-type proteins [26,27]. The lysine amino acid in the domain III of Nav1 channels enhances the selectivity for sodium [28,29]. Conversely, the glutamic acid found in Nav2 is characteristic of calcium channels. Site-directed mutagenesis studies in the cockroach, *Blattella germanica*, highlighted the likelihood that glutamic acid in domain III of *BSC1* gene plays a key role in the selectivity for calcium [30]. Similarly, in insects, the two genes *para* (DEKA) and *DSC1* (DEEA), were initially classified as sodium channels [12]. Recently, functional studies demonstrated that the *DSC1* gene could instead be a new type of voltage-gated cation channel [31]. A recent study proposed that the selectivity filter of choanoflagellates and metazoans (DEEA) is an intermediate between calcium channel (EEEE) and sodium channel (DEKA) and remains present in Nav2 of invertebrates [7]. These channels (DEEA) would be selective to both calcium and sodium ions [32]. Our results raise the issue of the nature and selectivity of *CgNav2* channel in the Pacific oyster. *CgNav2* could be a channel intermediate between the sodium and calcium channels. These uncertainties about the nature of *CgNav2* led the study to focus only on *CgNav1*, a member of the voltage-gated sodium channels known to be the target of PST.

### 3.2. *CgNav1* Genotype Could Confer a Certain Resistance of Oysters to PST

To investigate if the variability of PST accumulation between oysters is related to the existence of several forms of *CgNav1* with different sensitivity to PST, two hypotheses were explored: (i) genetic polymorphism in PST binding sites of *CgNav1* leads to different phenotypes of sensitivity to PST; (ii) alternative splicing of *CgNav1* produces protein isoforms with different sensitivity to PST.

DNA polymorphism analysis in *C. gigas* revealed that the 10 amino acids of the PST binding sites were strictly monomorphic, despite the high nucleotid polymorphism of *CgNav1*, similar to the global SNP level described for this species [33]. Nav are encoded by genes highly conserved through evolution. This probably reflects the critical functional role of these proteins in the regulation of excitability [34], in particular in the most conserved pore region, the critical zone for the selection of ions and their flow [16,35]. In softshell clams, PST resistance is conferred by the substitution of the glutamic acid into aspartic acid in the PST binding site of domain II of Nav channel [4]. For this species, exposure to PST constitutes a strong selection pressure because PST causes mortalities of sensitive clams, leading to the increase in the resistant allele in populations regularly exposed to *Alexandrium*. In the Pacific oyster, at the same position, a glutamine is observed for *CgNav1*. Sensitivity studies of rat Nav1.2 revealed that mutation E945Q in domain II leads to resistance to STX and decreases sodium conductance [17,36]. As a result, oysters could have some resistance to STX attributable to the glutamine, which is consistent with the absence of mortalities observed in Pacific oyster populations during *Alexandrium* blooms. The high frequency of this *CgNav1* “resistant” genotype in *C. gigas* populations could result from an ancestral polymorphism followed by selection under PST pressure in native Asian origins of the Pacific oyster, as the same genotype was found in the Japanese and French populations. This hypothesis needs to be confirmed by functional electrophysiological studies using heterologous expression of these genes in *Xenopus* oocytes as performed in insects [12].

### 3.3. *CgNav1* is Spliced in Tissue-Specific Variants

The three variants (A, B and C) characterized for *CgNav1* resulted from alternative splicing of the two exons 5 and 7, likely mutually exclusive because the complete form of the cDNA with exons 5 and 7 has never been detected. Our results showed that spliced exons were localised in the inter-domain region between domains I and II (ID I-II). This region is a common area of alternative splicing for voltage-gated sodium channels in many species, such as *Drosophila melanogaster* [37,38], *Musca domestica* [39], *Bombyx mori* [40], *Cancer borealis* [41], or in mammalian sodium channels [42,43]. In accordance with the role of Nav in excitable cells, *CgNav1* mRNA appeared to be expressed in nerve cells of the central nervous system of oysters composed of visceral and cerebral ganglia, as well as in the peripheral nervous system composed of nerves innervating the tissues, such as branchial (at the base of the gills) or circumpallial (running along the mantle edge) nerves. Awad et al. specified in a study on rat that Nav1 mRNAs distribution generally corresponds to the localisation of the protein they encode [44]. As a result, *CgNav1* mRNAs localization would translate *CgNav1* channel distribution, even if further studies on protein are needed to confirm this pattern. The variant B was expressed in all the tissues; nerve cells of nerves or ganglia were stained by ISH, but not in the other tissues. The *CgNav1B* could be a form of *CgNav1* specific to nerve cells. Conversely, the variant A was only expressed in muscles or in tissues with abundant muscular fibres: in the striated muscle, the mantle, and to a lesser extend in the smooth muscle and gills. Expression of *CgNav1* observed in muscles of oysters is likely in relation to the function of Nav channels in neuromuscular communication. In fact, Nav channels are found in neuromuscular junctions and participate in the propagation of the action potential in the postsynaptic membrane, allowing the contraction of the muscle. As a result, *CgNav1A* and *CgNav1B* could be the specific form to neuromuscular junctions and nerve cells, respectively. The shortest form of *CgNav1*, the variant C seemed to present ubiquitous expression.

### 3.4. Potentially Different Pathways of Regulation Exist for *CgNav1*

The variant B of *CgNav1* expressed in the nervous system of oysters encodes a larger protein than proteins translated from variants A and C. This variant results from the retention of the exon 7, encoding a larger ID I-II with 12 putative protein kinase A sites. The ID I-II is known to be an important region for protein regulation because of its richness in PKA phosphorylation sites. The alternative splicing of exons containing PKA sites allow conditional phosphorylation of the Nav channels. Smith and Goldin demonstrated in the rat that the ID I-II of Nav channel is longer in brain than in skeletal muscle, and that the brain channel had many phosphorylation sites involved in PKA signalling pathways [45]. Similarly, in oysters the results suggest that only variant B of *CgNav1* could be modulated by PKA. It is also consistent with the presence of variant B in the nervous system and not in striated muscle. Accordingly, alternative splicing could be a mechanism involved in regulation of sodium channel expression in oysters. Phosphorylation of sodium channels by PKA and protein kinase C (PKC) has been shown to reduce the peak sodium current and modulates activation and inactivation phases [45]. A study in the cockroach identified two types of sodium current with two different signalling pathways, one of which was phosphorylation by PKA [46]. In parallel, in the same species, two current types were proposed to originate from the alternative splicing of one Nav gene [47]. Otherwise, this could suggest that variant B of *CgNav1*, exclusively present in the nervous system of the Pacific oyster, could be regulated by the PKA pathway, but variants A and C would be regulated by other pathways. In the same way, the different splice variants of *CgNav1* could generate different current types. Functional studies of each form of *CgNav1* would allow the study of electrophysiological properties of these channels.

### 3.5. The Level of PST Accumulation Would Be Independent of *CgNav1* Transcription Level

The exposure of oysters to the PST-producer *A. minutum*, caused large individual variability of toxin accumulation in the digestive gland of oysters, as reported in a previous study [20]. The expression of the 3 variants of *CgNav1* has been analysed and compared individually to toxin content. The variability of PST accumulation did not appeared correlated to *Cg1* splice variant expression levels. However, in striated muscle, *CgNav1A* and *CgNav1C* expression tended to increase according to PST accumulation up to a threshold of 100 μg STX eq. 100 g^−1^ of wet digestive gland. This could suggests that when toxins bind to the channel and block the nerve impulses, the oyster has to compensate and produce more channels to maintain a sufficient flow of sodium for cell function. Studies revealed that treatment with Nav channel blockers (like PST) increases cell-surface expression of Nav channels [48]; however, in visceral ganglia, no correlation was observed between *CgNav1* (B and C) expression and toxin content. The activity of *CgNav1B* could be regulated at post-translational level by PKA, as proposed in the previous section, rather than by modification of transcription rate. The *CgNav1C*, the shortest and less-expressed form, seemed to be regulated as *CgNav1A* in striated muscle but not in visceral ganglia and could have tissue-specific regulation. In these experiments, oyster Nav channels seem to have an activity-dependent regulation to optimise activity and avoid hyper-excitability. When the toxin content in digestive gland exceeded 100 μg·100 g^−1^, the *CgNav1* mRNA synthesis in striated muscle did not increase further, possibly because of physiological disorders provoked by PST disturbing gene regulation processes. In oysters with the highest toxin content, ca. 900 μg STX eq. 100 g^−1^ of digestive gland, *CgNav1* expression was near zero. This could correspond to the impossibility for oysters to maintain neuromuscular communication, leading to paralysis.

In conclusion, the alternative splicing of *CgNav1* gene in Pacific oysters may be a critical mechanism allowing the production of *CgNav1* channels adapted to different nervous functions. Specific regulation of *CgNav1* isoforms may result in different channel properties. Given the absence of protein polymorphism at the PST binding site of specific variants in oysters accumulating the less or the more PST, we can rule out the initial hypothesis that different forms of *CgNav1* could explain the inter-individual variability of the PST accumulation. Our results suggest that the blocking of Nav by PST in oysters could trigger the activation of regulatory pathways to modulate Nav channel expression. The protein sequence of *CgNav1* would confer to all the forms of oyster Nav1 a relative resistance to STX. To validate this hypothesis, the specific sensitivity of each *CgNav1* variant to PST needs to be investigated, using, for example, heterologous expression in *Xenopus* oocytes and an electrophysiological approach. Accordingly, in Pacific oysters the quantity of PST accumulated would result from individual variability in ecophysiological capacities such as filtration, detoxification or even biological rhythms rather than differential sensitivity of Nav channel.

## 4. Materials and Methods

### 4.1. Phylogenetic Analyses of the Voltage-Gated Sodium Channel α Subunit of *Crassostrea gigas*

Amino acid sequences alignment was based upon a multiple alignment method using MAFFT 7 [49]. Alignments were refined to select reliably-aligned positions by using Gblocks version 0.91b [50]. The substitution model LG (+I +G +F) used in this study was selected using ProtTest 3.4.2 [51]. The maximum likelihood phylogenetic tree was constructed using PhyML 3.0 [52], and tree robustness was assessed with 100 bootstrap replications. Tree visualization was performed using FigTree v1.4.2 [53]. Sequences used for the analysis are presented in Table 4.

The oyster Nav2 was not studied for further experiments as its sequence suggested no selectivity for sodium ions only, meaning that it may not be a true Nav channel (see explanation in the result section).

### 4.2. Biological Material

#### 4.2.1. *Crassostrea gigas* Oysters

For polymorphism analyses (Section 4.6), a study was conducted on 4 populations of *C. gigas*. Three French populations located on the west coast: Bay of Brest, North Brittany; Larmor Baden, South Brittany; Ile de Ré, Charente-Maritime, and regularly exposed to toxic bloom of PST (*n* = 50 per population) and one Japanese population (*n* = 20) located in the Bay of Sendai, known to be exposed to toxic blooms of PST for many years [67]. Gills were sampled from oysters and stored in ethanol for DNA extraction. For the characterization of Nav cDNA sequences (Section 4.7) and expression analysis (Section 4.8 and Section 4.9), wild oysters were sampled in the Bay of Brest (Brittany, France). Nine different tissues were dissected from each individual: mantle, gills, heart, smooth muscle, striated muscle, labial palps, visceral ganglia, gonad, and digestive gland. Immediately after dissection, tissues were placed in RNA later solution (Invitrogen, Carlsbad, CA, USA) and stored at −80 °C until RNA extraction. For the study of *CgNav1* variant expression in experiments 1 and 2 (Section 4.3), oysters were obtained from the experimental hatchery of Ifremer in La Tremblade (Charente-Maritime, France), and from a shellfish farmer in the Bay of Arcachon (Gironde, France), respectively. Immediately after dissection, tissues were placed in RNA later solution (Invitrogen, Carlsbad, CA, USA) and stored at −80 °C until RNA extraction.

#### 4.2.2. Microalgae Cultures

The dinoflagellates *Alexandrium minutum* Halim (1960) strain Daoulas 1257 (isolated from Brest Bay, France) and strain AM89BM (isolated from Morlaix Bay, France) were used for toxic algal exposure (experiment 1 and experiment 2, respectively), and the non-toxic dinoflagellate *Heterocapsa triquetra* (Ehrenberg) Stein, strain HT99PZ (isolated from Penzé Bay, France) was used as a control. Both dinoflagellate cultures were grown in L1 medium [68] at 16 °C with a light/dark cycle of 12:12 h and were harvested during exponential growth phase. Algal cell densities were determined by counts using Nageotte counting chamber (PolyLabo, France) under a light microscope.

### 4.3. Experimental Design for Oyster Exposure to PST

To test the possible relationship between *CgNav1* expression and PST accumulation, oysters were exposed to the toxic *A. minutum* or the non-toxic dinoflagellate *Heterocapsa triquetra* similar in size and shape to *A. minutum*. Both experiments were set up in two different phases: an acclimation period of 7 days to the non-toxic dinoflagellate followed by an exposure period of 4 or 6 days to the toxic dinoflagellate species or to the control. Each tank was supplied with microalgae using a peristaltic pump. Central air-lifts were used to homogenize microalgal concentration and water in each tank.

#### 4.3.1. Experiment 1

Oysters were placed randomly in 18 L replicated tanks with 12 oysters per tank. During the acclimation period, all oysters were fed with a continuous flow of 19 L·day^−1^ of seawater with *H. triquetra* (10^6^ cells·L^−1^), then oysters were separated into two groups, exposed for 4 days to a continuous flow of 19 L·day^−1^ of seawater with *A. minutum* strain Daoulas 1257 (3.10^6^ cells·L^−1^; *n* = 60, 5 replicates of 12 individuals) or *H. triquetra* (10^6^ cells·L^−1^; *n* = 36, 3 replicates of 12 individuals). At the end of the exposure period, digestive glands of oysters were dissected, weighed, frozen, and stored in liquid nitrogen until toxin analyses. Visceral ganglia and striated muscle also were dissected and stored in RNA later solution (Invitrogen, Carlsbad, CA, USA) at −80 °C until mRNA expression analyses.

#### 4.3.2. Experiment 2

Oysters were distributed randomly into six tanks, with 29–30 oysters per tank. During the acclimation period, oysters were fed with a continuous flow of 144 L·day^−1^ of seawater with *H. triquetra* (10^5^ cells·L^−1^). Then oysters were separated into two groups exposed for 6 days to a continuous flow of 144 L·day^−1^ of seawater with *A. minutum* strain AM89BM (10^5^ cells·L^−1^; *n* = 88; 3 replicates) or *H. triquetra* (10^5^ cells·L^−1^; *n* = 88; 3 replicates). At the end of the exposure period, digestive glands, visceral ganglia, and striated muscle were sampled as in experiment 1.

### 4.4. Toxin Quantification by Liquid Chromatography/Fluorescence Detection

To extract the PST, 5 mL of 0.1 N hydrochloric acid were added, and the samples were mixed with a high-speed homogenizer (15,000 rpm) for 2 min. The pH was adjusted between 2.0 and 4.0, then the samples were centrifuged at 4200× *g* for 10 min at 4 °C. The supernatants were filtered on 10-kDa polyethersulfone (PES) filters, and the toxin content was analyzed using the liquid chromatography with fluorescence detection (LC/FD) PSP toxin analyses method of Van de Riet [69]. The toxins GTX, dc-GTX, dc-STX and STX were separated using a reverse chromatography column (Zorbax Bonus RP, 3.5 μM, 4.6 mm × 150 mm, Agilent Technologies, Massy, France) with a flow rate of 0.8 mL·min^−1^. The eluent pH and/or column temperature were optimized to separate dc-GTX3/GTX5/dc-GTX-2 and C1/C2. The toxin concentrations were determined using certified standards provided by CNRC (Halifax, NS, Canada).

### 4.5. DNA and RNA Extractions and cDNA Synthesis

Genomic DNA was extracted from oyster gills with the DNeasy Blood and Tissue kit (Qiagen, Germantown, MD, USA) according to the manufacturer’s instructions. The concentration and purity of DNA were analysed with a Nanodrop 8000 spectrophotometer (Thermo Scientific, Waltham, MA, USA). Total RNA was extracted using TRI Reagent^®^ (Sigma-Aldrich, St. Louis, MO, USA) following manufacturer’s instructions. Samples were treated with RTS DNase™ kit (MO BIO Laboratories, Germantown, MD, USA) to avoid genomic DNA contamination. The concentration and purity of all RNA were estimated with a Nanodrop 8000 spectrophotometer (Thermo Scientific, Waltham, MA, USA). RNA integrity was assessed by electrophoresis on agarose gel. cDNA synthesis was performed using 1 μg of total RNA primed with an Oligo(dT)_18_ and reverse-transcribed into first strand cDNA with the RevertAid H minus First Strand cDNA Synthesis kit (Fermentas, York, UK).

### 4.6. Single Nucleotid Polymorphism of *C. gigas* Nav1 α Subunit Gene

The DNA sequence of voltage-gated sodium channel α subunit (Nav1) of *C. gigas* was obtained from Zhang et al. [21]. This sequence (CGI_10001852) was first used as a reference sequence to design primers for DNA amplifications. The single nucleotid polymorphism of PST binding region was analysed by PCR amplification and sequencing (Figure 1). The region including the P segment of each domain of the Nav1, corresponding to the zone targeted by PST, was amplified by PCR with specific primer pairs (Table 5). Amplifications were performed in 25 μL of final reaction mixture. Each reaction contained 250 ng of DNA, 1.5 mM MgCl_2_, 200 μM dNTPs, 0.1 μM of each primer, 5 μL of Buffer 5× and 1.5 U polymerase GoTaq Flexi DNA (Promega, Madison, WI, USA). Cycling conditions were 2 min at 95 °C, 40 cycles of denaturation step for 45 s at 95 °C, annealing step for 45 s at 60 °C and elongation step for 45 s at 72 °C and a final step for 5 min at 72 °C. PCR products were verified by electrophoresis on agarose gel before sequencing (Sanger ABI 3730xl, GATC Biotech, Cologne, Germany). Chromatograms were checked and corrected by hand if needed and aligned to locate the SNP polymorphic site. A variation in the sequence was considered as a SNP only when its occurrence was above the threshold of 5% of the total number of oysters sampled. Nucleotide sequences then were translated into amino acid sequences to identify synonymous or non-synonymous mutations.

### 4.7. Amplification and Sequencing of the cDNA of *CgNav1* α Subunit

The full cDNA sequence was amplified step by step using the set of primers described in Table 5. The fragments “a” to “h” amplified to cover the full cDNA sequence are represented in Figure 1. A consensus sequence of the cDNA of *CgNav1* α subunit has been obtained from sequencing data using the Geneious software version 7.0.6 [70]. The fragments were amplified in 5 oysters and 9 tissues: striated and smooth muscles, mantle, visceral ganglia, heart, digestive gland, gills, gonad and labial palps. Amplification was performed in 50 μL of final reaction mixture. Each reaction contained 2 μL of cDNA, 1.5 mM MgCl_2_, 200 μM dNTPs, 0.1 μM of each primer (Table 5), 10 μL of Buffer 5× and 1.5 U polymerase GoTaq Flexi DNA (Promega, Madison, WI, USA). Cycling conditions were 2 min at 95 °C, 40 cycles of denaturation step for 45 s at 95 °C, annealing step for 45 s at 60 °C and elongation step for 4 min at 72 °C and a final step for 5 min at 72 °C. Specificity of amplification were checked on agarose electrophoresis and cloned into pCR4-TOPO vector with the TOPO TA cloning kit (Invitrogen) before sending to sequencing (Sanger ABI 3730xl). The characterization of splice variants was performed using PCR. Specific primers were designed to encompass the cytoplasmic loop linking domain I and domain II corresponding to fragment “c” which varies between variants.

### 4.8. Localization of *CgNav1* α Subunit mRNA Expression by In Situ Hybridization

The tissue localization of *CgNav1* mRNA was studied by in situ hybridization (ISH). For each individual, 4 transversal slices of 3 mm thickness were performed to study all sampled tissues: anterior region of the body (labial palps); anterior-middle region (digestive gland, gills, mantle, gonad); posterior-middle region (muscles, heart); posterior region (muscles, visceral ganglia). Samples were fixed immediately in Davidson’s fixative for 24 h at 4 °C, and then in ethanol 70% at 4 °C until inclusion, in RNAse free conditions. Tissues were dehydrated and embedded in paraffin wax using classical histological protocol according to Howard et al. [71], then were sectioned to 5 μm thickness with a microtome. Sections were mounted on glass slides treated with polylysine.

Specific riboprobes were designed from the cDNA sequence of *C. gigas* Nav9 (CGI_10001852). A 643 bp fragment, designed from the cDNA sequence (CGI_10001852) of *CgNav1*, was used as the template to synthesize riboprobes specific to all variants of *CgNav1* (Table 3). The amplified sequence was cloned into pCR4-TOPO vector with the TOPO TA cloning kit (Invitrogen, Carlsbad, CA, USA). Sense and antisense riboprobes were synthesized by in vitro transcription from linearized plasmids (MEGAscript kit, Ambion, Austin, TX, USA). Digoxigenin (DIG) labeling was performed by the incorporation of modified nucleotides UTP-DIG with the DIG RNA labeling kit (Roche Diagnostics GmbH, Mannheim, Germany).

The in situ hybridization protocol was adapted from Santerre et al. [72]. Briefly, tissue sample sections were treated with 10 μg/mL proteinase K (Sigma-Aldrich, St. Louis, MO, USA) at 37 °C for 10 min, post-fixed with 4% paraformaldehyde in PBS for 7 min and pre-hybridized with yeast tRNA for 1 h 30 min at 50 °C. Hybridization was then performed by incubating tissue sections with DIG-labeled sense or antisense riboprobes in hybridization buffer (50% formamide, 4× saline-sodium citrate buffer (SSC), 2 mM EDTA, 1% Denhardt’s solution, 10% dextran sulfate and 0.5 mg·mL^−1^ of yeast tRNA) for 16 h at 55 °C. After extensive washes with 50% formamide, 2× SSC at 45 °C, 2X SSC and 1× SSC at 37 °C, revelation was performed by incubating sections with anti-DIG antibody coupled to alkaline phosphatase (Roche Diagnostics GmbH, Mannheim, Germany) for 2 h at room temperature. Sections were then incubated in detection buffer (0.1 M Tris-HCl, 0.1 M NaCl) for 2–4 h in darkness. The mounting between slide and cover slip was performed with Canada balsam (Sigma-Aldrich, St. Louis, MO, USA). Sections were examined and photographed using an optical microscope (Leica DM-IRB, Nussloch, Germany). Hybridization with sense riboprobes was used as negative control.

### 4.9. Expression of *CgNav1* mRNA by Real-Time PCR

Expression of *CgNav1* transcripts was studied by real-time PCR in 8 tissues of 6 oysters. The specificity of primers was validated by sequencing of PCR product and analysis of the melting profile. Each amplification was performed using LightCycler 480 (Roche Diagnostics GmbH, Mannheim, Germany) in 10 μL final reaction mixture containing 5 μL of SYBR Green I Master Mix (2×) (Thermo Fisher Scientific, Waltham, MA, USA), 0.5 μL of each primer (10 μM) for Nav or reference genes *GAPDH* and *EF1α* (Table 3), 1 μL of cDNA, RNA DNase treated (negative control) or water (blank control) and 3 μL of free-RNase water. Cycling conditions were 10 min at 95 °C, 40 cycles of denaturation step for 10 s at 95 °C, annealing step for 20 s at 57 °C and elongation step for 15 s at 72 °C. All PCR reactions were run in triplicate and crossing point (Cp) values used were the mean of this replicates. The efficiency of amplification was calculated from the slope of the standard curve constructed with five serial dilutions of a pool of cDNA (calibrator) from striated muscle and visceral ganglia in equal proportion. The expression stability of the reference genes (*EF1α*, *GAPDH* and *Actin*) was tested using NormFinder (Version 0.953) algorithm as an Excel add-in. The best stability value (0.003) was obtained for the combination of *EF1α* and *GAPDH* genes. As a result, *CgNav1* transcript levels were normalised using the geometric mean of *GAPDH* and *EF1α* as reference genes and quantified with the E-method using the LightCycler 480 software 1.5.1.62 (Roche Diagnostics GmbH, Mannheim, Germany). mRNA levels of splice variants were expressed in relative quantity (Qr) compared to geometric mean of reference genes.

### 4.10. Data Analysis

BLAST searches were performed online at NCBI BLAST [73]. Multiple sequence alignments were carried out in Clustal Omega [74]. Exon-intron structure of sequences was assessed by aligning mRNA sequences to genomic sequences with the program MGAlign [75]. Donor and acceptor sites were predicted using a splice site predictor, NNSPLICE version 0.9 [76]. The deduced amino acid sequences were calculated by the ExPASy Proteomics Server [77]. Analyses of the predicted 2D structure of protein isoform were performed with the HCA program (Hydrophobic Cluster Analysis) from Mobyle portal [78]. Phosphorylation sites were predicted by NetPhos 3.1 server [79] and protein kinase A (PKA) and protein kinase C (PKC) sites were predicted with KinasePhos 2.0 server [80] and pkaPS server [81]. All statistical analyses were executed with R 3.2.2 [82]. The validation of the optimal reference gene for the normalization of real-time PCR data was performed using the NormFinder version 0.953 algorithm as an Excel add-in. Log (natural) transformed Cp values were used as input data. The analysis of the reference genes stability was completed by multiple-comparison test with Tukey’s HSD method. Comparisons of mRNA levels (relative to geometric mean of *EF1α* and *GAPDH* mRNA) between splice variants were performed with a Kruskal-Wallis test, followed by a non-parametric post-hoc with the Wilcoxon signed rank test. Pearson’s correlation was applied to assess relation between toxins accumulation and Nav mRNA expression. No correlation was applied when samples are less than three.

## Figures and Tables

**Table 1:** Analysis of the nucleotid polymorphism of regions including PST binding site for each domain (DI to DIV) of *C. gigas* Nav1 α subunit.

Domains	Populations	N	Pe	Pi	Le	Li	∏e	∏i	∏t
DI	LOG	15	1	3	117	311	0.009	0.010	0.009
	LB	12	1	2	117	311	0.009	0.060	0.007
	RE	20	1	3	117	311	0.009	0.010	0.009
	JAP	13	1	3	117	311	0.009	0.010	0.009
DII	LOG	48	5	-	136	-	0.037	-	0.037
	LB	46	5	-	136	-	0.037	-	0.037
	RE	42	5	-	136	-	0.037	-	0.037
	JAP	19	6	-	136	-	0.044	-	0.044
DIII	LOG	14	0	7	140	188	0	0.037	0.021
	LB	8	0	6	140	188	0	0.032	0.018
	RE	11	0	5	140	188	0	0.027	0.015
	JAP	7	0	5	140	188	0	0.027	0.015
DIV	LOG	26	4	-	301	-	0.013	-	0.013
	LB	25	5	-	301	-	0.017	-	0.017
	RE	25	5	-	301	-	0.017	-	0.017
	JAP	20	4	-	301	-	0.013	-	0.013

LOG: Logonna-Daoulas, LB: Larmor Baden, RE: St Clément des Baleines, JAP: Japan. N: number of oyster analysed, Pe: number of SNP in exon, Pi: number of SNP in intron, Le: exon length (bp), Li: intron length (bp), ∏e: number of SNP per coding sites (Pe/Le), ∏i: number of SNP per non-coding sites (Pi/Li), ∏t: total number of SNP per sites ((Pe + Pi)/(Le + Li)), dash: no data.

**Table 2:** Structural characteristics of splice variants of *C. gigas* Nav1 α subunit.

Name of Sequences	Total cDNA Size (bp)	Total Predicted Protein Size (aa)	Alternatively Spliced Fragments	Spliced cDNA Fragments Size (bp)	Spliced Protein Fragments Size (aa)
CGI_10001852	5205	1734	/	/	/
Variant A	5532	1844	−exon 7	−405	−135
			+exon 15	+33	+11
Variant B	5877	1959	−exon 5	−27	−9
Variant C	5505	1835	−exons 5 and 7	−432	−144
			+exon 15	+33	+11

Base pairs, bp; aa, amino acids.

**Table 3:** Primers used for in situ hybridization and real-time PCR. Accession number: GAPDH, XM_011446602 [23], EF1α, AB122066.

Amplicon Names	Primer Names	Primer Sequences (5′–3′)	Length (bp)
*Primers used for the* in situ *hybridization*			
Exon 24	*CgNav9*_e25F	AGGCGGGTGTTATGTTCTTG	20
	*CgNav9*_e25R	GCGGTATCTTCGTGAATGGT	20
*Primers used in splice variants real-time PCR*			
Variant A	*CgNav9*_a5F	CTCTTGTGCTCATTCCAGCA	20
	*CgNav9*_s7R	GACCCATTTATTGACCCCTTCT	22
Variant B	*CgNav9*_s5F	CGAAAGATTCAACAAACAATGCATG	25
	*CgNav9*_a7R1	TTAAAGGTTGATGGTCAGCGTGATT	25
Variant C	*CgNav9*_s5F	CGAAAGATTCAACAAACAATGCATG	25
	*CgNav9*_s7R	GACCCATTTATTGACCCCTTCT	22
Variant D	*CgNav9*_a5F	CTCTTGTGCTCATTCCAGCA	20
	*CgNav9*_a7R1	TTAAAGGTTGATGGTCAGCGTGATT	25
GAPDH	qFw_GAPDH	GGAGACAAGCGAAGCAGCAT	20
	qRev_GAPDH	CACAAAATTGTCATTCAAGGCAAT	24
EF1α	qfElongN	GATTGCCACACTGCTCACAT	20
	qrElongN	AGCATCTCCGTTCTTGATGC	20

**Table 4:** Protein sequences of Nav channel α subunit used for the phylogenetic tree construction.

Species Name	Nav Name	Common Name	GenBank Accession Number	Size (aa)	Reference
*Cyanea capillata*	*CcNav*	Lion’s mane jellyfish	AAA75572	1740	[54]
*Polyorchis penicillatus*	*PpNav*	Hydrozoan jellyfish	AAC09306	1695	[55]
*Aiptasia pallida*	*ApNav*	Pale anemone	AAB96953	1810	[56]
*Capitella teleta*	*CtNav2*	Polychaete worm	JGI 134859 *	1694	
*Crassostrea gigas*	*CgNav2* (Nav5)	Pacific oyster	EKC21550	1986	[21]
*Blattella germanica*	*BgNav2* (BSC1)	German cockroach	AAK01090	2304	[57]
*Danaus plexippus*	*DpNav2*	Monarch butterfly	EHJ64356	1991	[58]
*Drosophila melanogaster*	*DmNav2* (DSC1)	Fruit fly	ABF70206	2409	[59]
*Cynops pyrrhogaster*	*CpNav*	Japanese common newt	AAD17315	2007	[60]
*Capitella teleta*	*CtNav1*	Polychaete worm	JGI 210954 *	1690	
*Doryteuthis opalescens*	*DoNav*	Opalescent inshore squid	AAA16202	1784	[61]
*Aplysia californica*	*AcNav1*	California sea hare	NP_001191637	1993	[62]
*Mya arenaria*	*MaNav*	Softshell clam	AAX14719	1435	[4]
*Crassostrea gigas*	*CgNav1* (Nav9)	Pacific oyster	EKC22630	1734	[21]
*Cancer borealis*	*CbNav*	Jonah crab	ABL10360	1989	[63]
*Blattella germanica*	*BgNav1* (para)	German cockroach	AAC47483	2031	[64]
*Danaus plexippus*	*DpNav1* (para)	Monarch butterfly	EHJ74501	2112	[58]
*Drosophila melanogaster*	*DmNav1* (para)	Fruit fly	AAB59195	2131	[65]
*Aedes aegypti*	*AaNav1* (para)	Yellow fever mosquito	ACB37023	2140	[66]
*Culex pipiens pallens*	*CpNav1* (para)	Northern house mosquito	AGO33659	2043	
*Culex quinquefasciatus*	*CqNav1* (para)	Southern house mosquito	AGO33660	2052	

* Predicted protein sequences from JGI Genome portal.

**Table 5:** Primers used for the amplification of PST binding site for domains I–IV and full length cDNA of *CgNav1* α subunit in *C. gigas* oyster.

Amplicon Names	Primer Names	Primer Sequences (5′–3′)	Length (bp)	Amplicon Size (bp)
*Primers used for segment P region amplification*				
DI	*CgNav9*_1f	TGACACTCACACAAACCCAGA	21	491
	*CgNav9*_1r	AACGAGCCCAGCAGTATCAC	20	
DII	*CgNav9*_2f	TGTTCTTGCCATTGTGGTGT	20	214
	*CgNav9*_2r	AAAGAACGGGACACAGATCG	20	
DIII	*CgNav9*_3f’2	GGTGTGCCTCATTTTCTGGT	20	385
	*CgNav9*_3r’2	CTGCACCGATATTCTCAGCA	20	
DIV	*CgNav9*_4f	GACGTCATGGACCAATTCCT	20	353
	*CgNav9*_4r	TTACAACCCTCCTCGTTCGT	20	
*Primers used for the amplification of full length cDNA*				
a	*CgNav9*_TF2	GCTGTGTACGACTAAAATGGAG	22	425
	*CgNav9*_e1R	ACGCGCTGAATAATGGATG	19	
b	*CgNav9*_Ch2F	AGCCCCTTTAACCCACTCAG	20	863
	*CgNav9*_1R	AACGAGCCCAGCAGTATCAC	20	
c	*CgNav9*_Ch2F	AGCCCCTTTAACCCACTCAG	20	1873
	*CgNav9*_Ch4R	CAAAAGCATCCAACACGATG	20	
d	*CgNav9*_Ch5F	AGCGACTACCTTCCTTTCGAG	21	984
	*CgNav9*_2Rs	GCTTGGTTCTCTCTCGTTCG	20	
e	*CgNav9*_Ch6F	GGAAGATGGGTCAAAGTCAAAG	22	1172
	*CgNav9*_3r’2	GCGTCATTCATTACTTCGATCC	22	
f	*CgNav9*_Ch8F	CCTGAATCTGTTCATCGGTGT	21	801
	*CgNav9*_4R	TTACAACCCTCCTCGTTCGT	20	
g	*CgNav9*_Ch9F	CACGTTCGGGATGAGTTTCT	20	867
	*CgNav9*_e25R	GCGGTATCTTCGTGAATGGT	20	
h	*CgNav9*_Ch10F	ACTACGCCGCAAGGGTTAT	19	590
	*CgNav9*_TR	GGGTTGATAACAGTGGGTGAA	21	
